# Peritoneal Tumor Carcinomatosis: Pharmacological Targeting with Hyaluronan-Based Bioconjugates Overcomes Therapeutic Indications of Current Drugs

**DOI:** 10.1371/journal.pone.0112240

**Published:** 2014-11-10

**Authors:** Isabella Monia Montagner, Anna Merlo, Gaia Zuccolotto, Davide Renier, Monica Campisi, Gianfranco Pasut, Paola Zanovello, Antonio Rosato

**Affiliations:** 1 Veneto Institute of Oncology IOV - IRCCS, Padua, Italy; 2 Department of Medicine, University of Padua, Padua, Italy; 3 Fidia Farmaceutici S.p.A., Abano Terme, Italy; 4 Department of Pharmaceutical and Pharmacological Sciences, University of Padua, Padua, Italy; 5 Department of Surgery, Oncology and Gastroenterology, University of Padua, Padua, Italy; Columbia University, United States of America

## Abstract

Peritoneal carcinomatosis still lacks reliable therapeutic options. We aimed at testing a drug delivery strategy allowing a controlled release of cytotoxic molecules and selective targeting of tumor cells. We comparatively assessed the efficacy of a loco-regional intraperitoneal treatment in immunocompromised mice with bioconjugates formed by chemical linking of paclitaxel or SN-38 to hyaluronan, against three models of peritoneal carcinomatosis derived from human colorectal, gastric and esophageal tumor cell xenografts. *In vitro*, bioconjugates were selectively internalized through mechanisms largely dependent on interaction with the CD44 receptor and caveolin-mediated endocytosis, which led to accumulation of compounds into lysosomes of tumor cells. Moreover, they inhibited tumor growth comparably to free drugs. *In vivo*, efficacy of bioconjugates or free drugs against luciferase-transduced tumor cells was assessed by bioluminescence optical imaging, and by recording mice survival. The intraperitoneal administration of bioconjugates in tumor-bearing mice exerted overlapping or improved therapeutic efficacy compared with unconjugated drugs. Overall, drug conjugation to hyaluronan significantly improved the profiles of *in vivo* tolerability and widened the field of application of existing drugs, over their formal approval or current use. Therefore, this approach can be envisaged as a promising therapeutic strategy for loco-regional treatment of peritoneal carcinomatosis.

## Introduction

Colorectal (CRC) and gastric cancers are the second and third most common causes of cancer-related death worldwide, respectively [1], [2]. Both tumor histotypes frequently spread in the peritoneal cavity, thus causing peritoneal carcinomatosis (PC) even in the early phase of the disease [3]–[5]. Patients with peritoneally diffused disease have a poor prognosis, with median survival of just few months [3]–[5]. Esophageal carcinoma is the sixth most common cancer in the world, with a long-term survival rate of only 27–41% [6]. In the advanced stages, this type of carcinoma shows extensive peritoneal tumor spread [7].

Once established, PC can be essentially regarded as a terminal clinical condition that is poorly amenable to further chemotherapeutic aggression [8]. Nonetheless, the last years have witnessed new therapeutic treatments based on cytoreductive surgery combined with intraperitoneal chemotherapy under hyperthermic condition, to produce a loco-regional control of peritoneal metastasis and to improve long-term survival of patients [9], [10]. This approach, however, requires the development of more efficient and less toxic chemotherapeutic agents [11].

Macromolecular drug delivery systems have been proposed as advanced approaches for improving antitumor treatments, especially with the aim of overcoming drug resistance, water insolubility, lack of selectivity and increasing tolerability [12]. Polymeric conjugates of chemotherapy agents are largely based on biocompatible polymers like dextran [13], [14], synthetic poly(L-glutamic acid) polymers [15], polyanhydrides [16], N-(2-hydroxypropyl)methacrylamide copolymers [17], [18], poly(ethyleneglycol) (PEG) [19], [20] and hyaluronan (HA), as reviewed by Duncan *et al*. [12]. HA [21], [22] is a linear polysaccharide that is ubiquitously distributed in the extracellular matrix, the synovial fluid of joints and the cartilage. By promoting cell motility, adhesion and proliferation, HA plays a pivotal role in biological processes as morphogenesis, wound repair, inflammation and cancer metastasis [23], [24]. These phenomena rely on the interaction with several receptors, among which the most representative are CD44 [25], [26], the receptor for hyaluronan-mediated cell motility (RHAMM, CD168) [24], and HARE (HA receptor for endocytosis) [27]. Since CD44 and RHAMM are overexpressed in a wide variety of cancers, including colorectal [11], gastric [28] and esophageal carcinoma [29], hyaluronan-drug bioconjugates should present a markedly enhanced selectivity for cancerous cells. Therefore, such approach can be envisaged to achieve an enhanced targeting of tumor tissue, and to prolong the retention of drugs within the body, besides providing advantages in drug solubilization, stabilization, localization and controlled release. In this regard, HA has already been conjugated to different antineoplastic drugs, generating new compounds with promising antitumor effects toward a broad panel of tumor histotypes [11], [21], [30]–[32].

Here, we compared the therapeutic effectiveness of two bioconjugates derived from the chemical linking of paclitaxel [21] or SN-38 [11], the active metabolite of irinotecan, to HA against three models of human PC xenografts in immunocompromised mice. We show that they performed successfully after intraperitoneal administration, having a comparable or enhanced therapeutic efficacy profile respect to free drugs, thus supporting their testing in clinical trials.

## Materials and Methods

### Drugs

The hyaluronan-based paclitaxel (ONCOFID-P) [21] and SN-38 (ONCOFID-S) [11] bioconjugates have been previously described. The batch of ONCOFID-S used was characterized by a SN-38 loading of 9.4%. Paclitaxel (Taxol) was from Bristol-Myers Squibb Italia (Rome, Italy), while irinotecan (CPT-11) and SN-38 were purchased from Antibioticos (Rodano, Italy). When needed, bioconjugates were labeled with the fluorochrome BODIPY TR cadaverine (Invitrogen, San Giuliano Milanese, Italy), as previously described [33].

### Tumor cell lines

The following human tumor cell lines were used: HCT-15 [34], HT-29 [35] and LoVo [36], colorectal adenocarcinoma; OE-33 [37], esophageal adenocarcinoma; KYSE-30 [38] and OE-21 [37], esophageal squamous carcinoma; MKN-45 [39], gastric adenocarcinoma. A CD44^high^ subpopulation of HCT-15 was isolated by immunomagnetic sorting, as previously reported [40]. Cells were grown in RPMI 1640 (EuroClone, Milan, Italy) supplemented with 10% (v/v) heat-inactivated fetal bovine serum (Gibco BRL, Paisley, UK), 2 mM L-glutamine (Gibco BRL), 10 mM HEPES (PAA Laboratories, Linz, Austria), 200 U/mL penicillin (Pharmacia & Upjohn, Milan, Italy), 200 U/mL streptomycin (Bristol-Myers Squibb Italia) and 1 mM sodium pyruvate (Lonza, Basel, Switzerland; not added in the case of MKN-45 cells), hereafter referred as to complete medium. Cell lines were maintained at 37°C in a humidified atmosphere containing 5% CO_2_.

### Cytotoxicity assay

The *in vitro* cytotoxicity of free drugs, ONCOFID-P, ONCOFID-S and fluorochrome-labeled bioconjugates was assessed against all cell lines using the ATPlite luminescence adenosine triphosphate (ATP) detection assay system (PerkinElmer, Zaventem, Belgium) [41], according to the manufacturer's instructions. Briefly, cells were resuspended in complete medium and seeded into 96-well flat-bottomed plates (8×10^3^/well); the day after, different drug concentrations were added (final volume, 100 µL/well) for 72 hours. At day 4, 50 µL of lysis solution were added to each well followed by addition of 50 µL of substrate solution and final counting of luminescence by the TopCount Microplate Counter (PerkinElmer). Within each experiment, determinations were performed in triplicate and experiments were repeated 5 times for each cell line. The percentage of cell survival was calculated by determining the counts per second (cps) values according to the formula: [(cps_tested_ - cps_blank_)/(cps_untreated control_ - cps_blank_)]×100, with cps_blank_ referring to the cps of wells that contained only medium and ATPlite solution. IC_50_ values were calculated from semi-logarithmic dose-response curves by linear interpolation.

### Flow cytometry analysis

CD44 and CD168 (RHAMM) expression in all tumor cell lines was evaluated by flow cytometry, as previously reported [33]. Interaction of bioconjugates with tumor cells was evaluated by incubating 3×10^5^ cell/sample in 1 mL of medium containing BODIPY-labeled ONCOFID-P (50 µg/mL in paclitaxel equivalents) or ONCOFID-S (50 µg/mL in SN-38 equivalents) at 37°C. At different time points thereafter (0.5, 1, 2, 5, 10, 15, 30 or 60 minutes), cells were harvested as previously described [21] and the fluorescence was compared with that of untreated cells. Where indicated, tumor cells were treated with BODIPY-labeled ONCOFID-P, and then with hyaluronidase (HA:hyaluronidase molar ratio of 25∶1 w/w; Sigma-Aldrich) for 4 hours at 37°C in PBS to remove non-internalized conjugates, before flow cytometry analysis. To evaluate the role of CD44 receptor in the interaction of ONCOFID-P with cancer cell lines, cells were incubated with BODIPY-labeled bioconjugate for 30 minutes in the presence of an anti-CD44 blocking mAb (10 µg/ml, clone 5F12, Lifespan Biosciences, Seattle, WA).

### Chemical inhibitors of endocytosis

To dissect the endocytosis pathway involved in cellular entry of bioconjugates, tumor cells were treated with different chemical inhibitors for 1 hour at 37°C in RPMI before drug exposure and hyaluronidase treatment, as reported above. The following inhibitors were used: amiloride (inhibitor of phagocytosis/micropinocytosis; 50 µM), chlorpromazine (inhibitor of clathrin-dependent endocytosis; 20 µg/ml), cytochalasin D (inhibitor of phagocytosis/micropinocytosis; 10 µg/ml), and filipin III (inhibitor of clathrin-independent, caveolin-mediated endocytosis; 10 µg/ml), all purchased from Sigma-Aldrich.

### Confocal microscopy analysis

To assess cell internalization of bioconjugates by confocal microscopy, tumor cell lines were seeded on glass coverslips in 12-well tissue culture plates at the concentration of 5×10^5^ cells/well. After 24 hours of culture, cells were incubated with BODIPY-labeled bioconjugates for 1 hour at 37°C, and then fixed for 20 minutes in 4% formaldehyde. Sample analysis was carried out with a Zeiss LSM 510 microscope (Carl Zeiss, Jena, Germany), using a long pass 560 nm filter. Lysosomal colocalization and microtubular visualization studies were carried out as previously described [40].

### Assessment of Topoisomerase I activity by plasmid relaxation assay

Topoisomerase I (Topo I) was isolated from tumor cell lines by Qproteome Nuclear Protein Kit (Qiagen, Milan, Italy), after incubation of cells (5×10^6^/sample) with ONCOFID-S (50 µg/mL in SN-38 equivalents), SN-38 (50 µg/mL) or complete medium (untreated cells) at 37°C for 1 hour. Enzyme activity was assessed using the Human Topo I Assay Kit for cell extracts (Inspiralis, Norwich, United Kingdom). Dilutions of cell extracts (1∶5, 1∶10, 1∶50, 1∶100 and 1∶500) were incubated for 30 minutes at 37°C with the relaxation mix containing a supercoiled DNA substrate (pBR322). Reaction was stopped by adding an equal volume of chloroform/isoamyl alcohol (24∶1). Samples were fractionated by 0.8% agarose gel electrophoresis, visualized by ethidium bromide staining and quantified by UV densitometry using the supercoiled and relaxed pBR322 plasmid as positive or negative control, respectively. Inhibition of Topo I activity was calculated as the ratio between the supercoiled fractions in treated cells and the positive control and expressed as percentage.

### Mice

Six to eight week-old female severe combined immunodeficiency (SCID) mice were purchased from Charles River Laboratories (Calco, Italy), and housed in our Specific Pathogen Free (SPF) animal facility.

### *In vivo* experiments and optical imaging

SCID mice were inoculated i.p. with 1×10^6^ HT-29, MKN-45 or OE-21 tumor cells. Pharmacological treatments were started at day 7 from tumor injection and carried out according to a q7dx3 schedule (every 7 days for 3 doses). Each experiment comprised groups of animals (six mice/group) that received ONCOFID-P i.p. (40 mg/kg in paclitaxel equivalents), or ONCOFID-S i.p. (19.2 mg/kg in SN-38 equivalents), or paclitaxel i.p. (10 mg/kg), or CPT-11 i.p. (60 mg/kg), or paclitaxel i.v. (20 mg/kg) or CPT-11 i.v. (100 mg/kg). Injected tumor cells had been previously transduced with a lentiviral vector coding for the firefly luciferase reporter gene [42] to track tumor growth *in vivo*. Bioluminescence (BLI) images were acquired at different time points after *in vivo* cell injection using the IVIS Lumina II Imaging System (PerkinElmer). Ten minutes before each imaging session, animals were anesthetized with isoflurane/oxygen and administered i.p. with 150 mg/kg of D-luciferin (PerkinElmer) in DPBS. A constant region of interest (ROI) was manually selected around the abdomen of animals and the signal intensity was measured as radiance (photon/sec) using the LivingImage software 3.2 (PerkinElmer). Tumor growth and response to therapy were monitored by BLI and by recording survival. Procedures involving animals and their care were in conformity with institutional guidelines (D.L. 116/92 and subsequent implementing circulars), and experimental protocols (project ID: 3/2012) were approved by the local Ethical Committee of Padua University (CEASA). During *in vivo* experiments, animals in all experimental groups were examined daily for a decrease in physical activity and other signs of disease or drug toxicity; severely ill animals were euthanized by carbon dioxide overdose.

### Statistical analysis

Survival curves and probabilities were estimated using the Kaplan-Meier technique. A log-rank test for comparisons, an Anova test or a Mann-Whitney Rank Sum Test were used when required. Analysis of data were done using the MedCalc (version 12) and SigmaPlot (version 12.3) statistical packages.

## Results

### HA receptor expression on target cancer cell lines

CD44 and CD168 are regarded as important receptors for hyaluronan binding. To assess their expression on colorectal, esophageal and gastric tumor cell lines, flow cytometry analysis was carried out. Results showed that CD44 was intensely expressed on all cell lines examined but HCT-15, which disclosed a weak positivity (about 20% of population; Fig. S1, inset). This cell line was immunomagnetically sorted in two subpopulations expressing the relevant marker at high and low intensity (Fig. S1A), to be further analyzed and compared with the parental cell line for sensitivity to the conjugated drugs (see below). RHAMM expression was more erratic and exclusively intracellular (Fig. S1B), being membrane levels of the receptor almost undetectable (data not shown).

### Analysis of interaction of bioconjugates with target cancer cell lines

**Figure 1:**
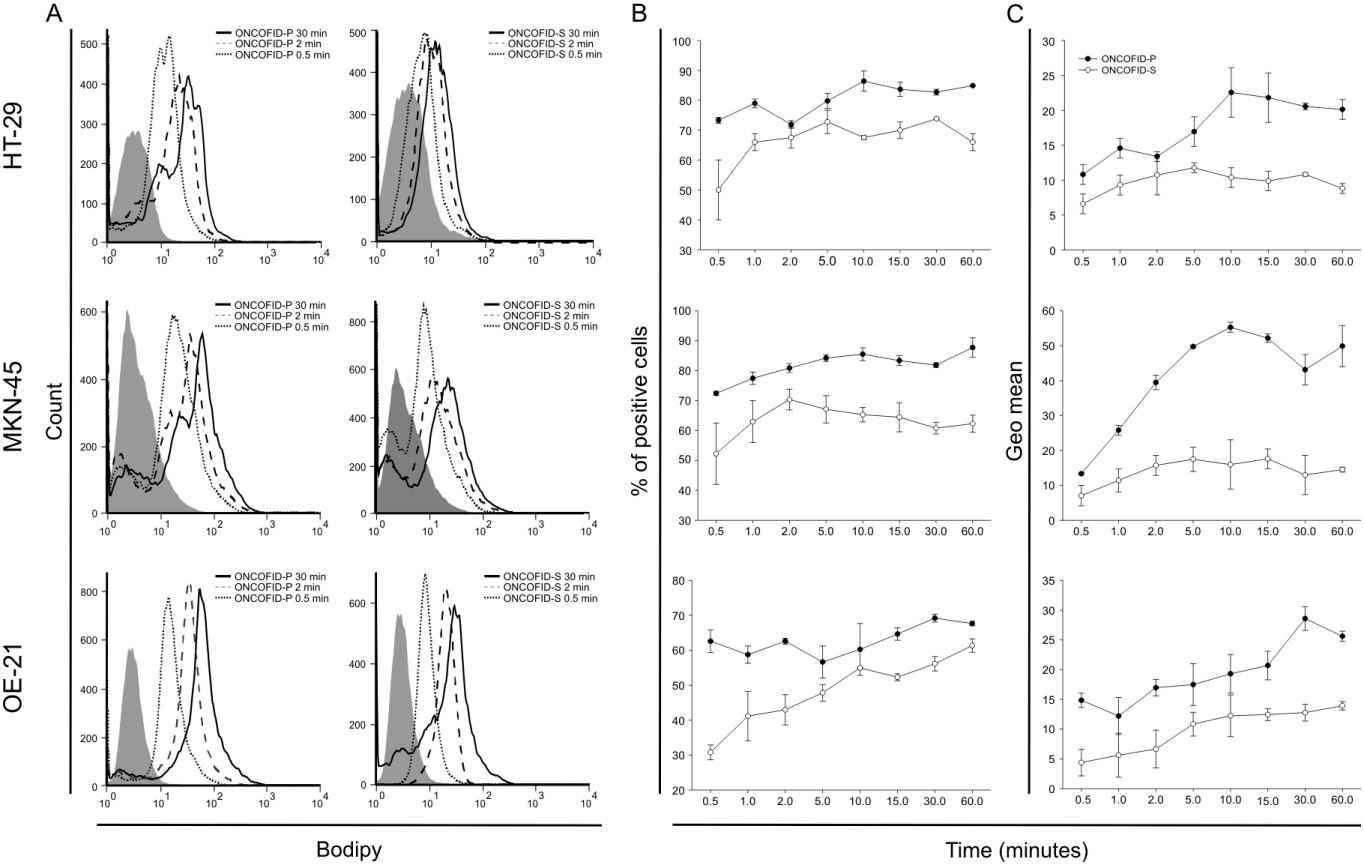
Interaction of bioconjugates with cancer cell lines. A, BODIPY-labeled ONCOFID-P (50 µg/mL in paclitaxel equivalents) or ONCOFID-S (50 µg/mL in SN-38 equivalents) were added to tumor cells and flow cytometry analysis was performed at different time points thereafter (0.5, 1, 2, 5, 10, 15, 30 or 60 minutes). Panels illustrate cytometry profiles at 3 representative time points. B, whole kinetics of interaction at all time points tested. C, kinetics of the fluorescence intensity (geo mean) detected on tumor cells at the same time points analysed as in B. Panels B and C report mean ± SD of 3 independent experiments.

To assess the direct interaction of ONCOFID-P and ONCOFID-S with CD44 expressing target cells, bioconjugates were labeled with the BODIPY fluorophore, incubated with tumor lines and analyzed cytofluorimetrically at different time points. Results disclosed that the bioconjugates readily bound to target cancer cells in a time-dependent manner (Fig. 1A and B). Indeed, the percentage of positive cells was already sensibly high just after 30 seconds, and progressively increased over time along with fluorescence intensity (Fig. 1C). To assess whether the fluorescent signal was simply due to a physical association to the cell membrane or truly reflected the internalized compound, the potentially non-internalized bioconjugates were removed by hyaluronidase treatment. For this and the following set of experiments, ONCOFID-P was selected as a prototype compound, since the critical moiety involved in cell interaction for both conjugates is represented by HA with the same MW and characteristics, and therefore results obtained with one can reliably apply also to the other bioconjugate. Results disclosed that such treatment slightly impacted the kinetics of physical binding between ONCOFID-P and tumor cells, as these latter readily became positive both in the presence or in the absence of hyaluronidase. On the other hand, removal of the membrane-bound labeled compound strongly reduced the fluorescence signal intensity and disclosed that a plateau was reached very rapidly, thus indicating that most of the bioconjugate was internalized in the first few minutes of interaction (Fig. S2).

Moreover, tumor cells were treated with a blocking anti-CD44 mAb to disclose the relevance of this receptor in the interaction of HA-conjugated drugs with target cells. While the fluorescent signal turned out to be strongly reduced, treatment with the blocking antibody did not completely abrogate the binding to the cells, thus suggesting a prominent but not exclusive role of CD44 in the uptake of the HA-conjugated compound ([24]; Fig. S3).

**Figure 2:**
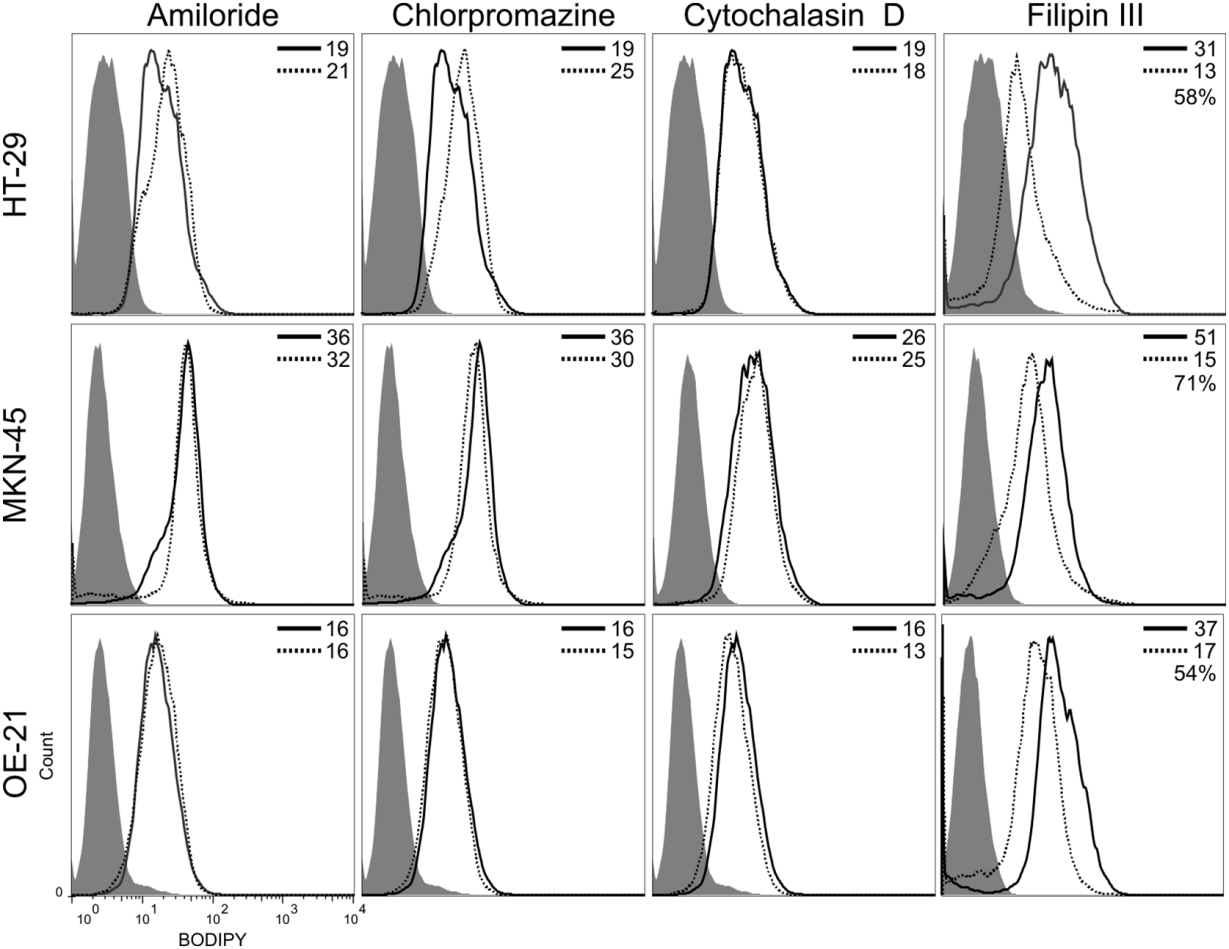
Endocytosis pathways involved in bioconjugate cell entry. HT-29, MKN-45 and OE-21 tumor cells were left untreated (solid line) or treated (dashed line) for 1 hour with selective chemical inhibitors of different pathways involved in endocytosis (amiloride, chlorpromazine, cytochalasin D and filipin III). Subsequently, cells were exposed for 30 minutes to ONCOFID-P and then treated with hyaluronidase for 4 hours, to be finally analyzed by flow cytometry. Data at the upper-right corner of each panel report the respective geo mean values, and the percentage of reduction induced by treatment.

**Figure 3:**
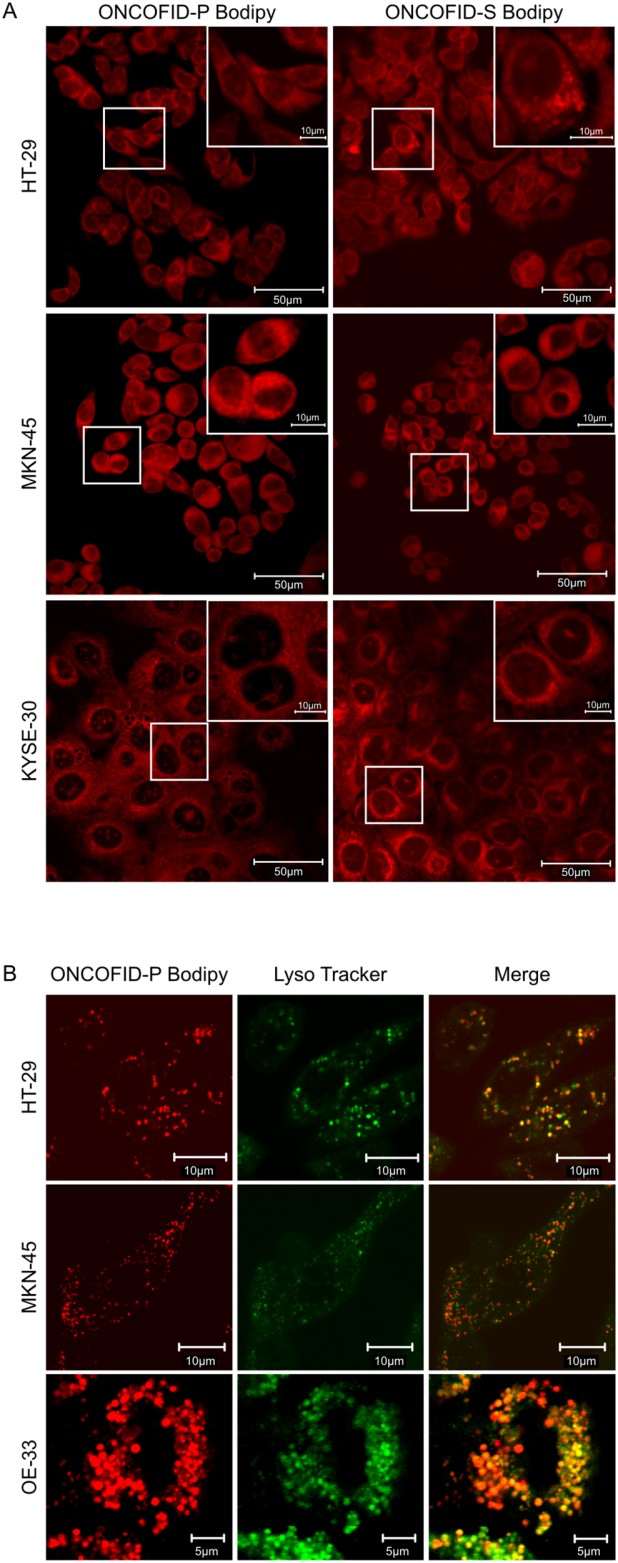
Confocal microscopy analysis and co-localization studies. A, accumulation of bioconjugates in HT-29, MKN-45 and KYSE-30. Cells were incubated with BODIPY-labeled ONCOFID-P (50 µg/mL in paclitaxel equivalents) or ONCOFID-S (50 µg/mL in SN-38 equivalents) for 1 hour, washed and fixed before analysis. B, co-localization analysis of bioconjugates in lysosomes. HT-29, MKN-45 and OE-33 cells were treated with LysoTracker green, incubated with BODIPY-labeled compounds and finally analyzed by confocal microscopy. Left pictures show the fluorescence of the labeled bioconjugates (red) in single cells, while central pictures illustrate signals (green) from lysosomes. The merging of the 2 components is visible in right pictures. Lysosomes were occupied by bioconjugates by ∼90% to 100%, as assessed by the Zeiss’profile software tool. Experiments were repeated at least twice with consistent results.

To dissect the different mechanisms of trafficking across the plasma membrane, tumor cells were treated with chemical agents that selectively block specific endocytic pathways. Amiloride, chlorpromazine and cytochalasin D leaved the internalization of ONCOFID-P substantially unaffected, thus ruling out phagocytosis/micropinocytosis and clathrin-mediated endocytosis as mechanisms for cellular entry. Conversely, the treatment with filipin III strongly reduced the intensity of fluorescence signal (Fig. 2), thus indicating that a clathrin-independent, caveolin-mediated endocytic pathway represents the major mechanism of internalization for HA-conjugates, in agreement with previously published data [43]. Overall, these data suggest that the bioconjugates physically associate to the cells, to be subsequently sequestered and accumulated. Indeed, this latter aspect was formally demonstrated by confocal microscopy analysis (Fig. 3A), which disclosed a relevant cytoplasmic accumulation. Moreover, bioconjugates appeared undergoing a rapid compartmentalization into discrete subcellular sites that were strongly reminiscent of lysosomes, as a similar pattern of fluorescence was observed by labeling tumor cells with a green fluorescent lysosome tracker. In fact, fluorescence signals derived from cells treated with the labeled compounds and the lysosome tracker completely overlapped (Fig. 3B and data not shown), thus demonstrating that bioconjugates did actually accumulate into the lysosomal compartments. Fluorochrome labeling of compounds did not modify their biological activity, as the fluorescent bioconjugates fully retained the cytotoxic potential (Fig. S4 and data not shown).

### Assessment of the mechanism of action of bioconjugates

**Figure 4:**
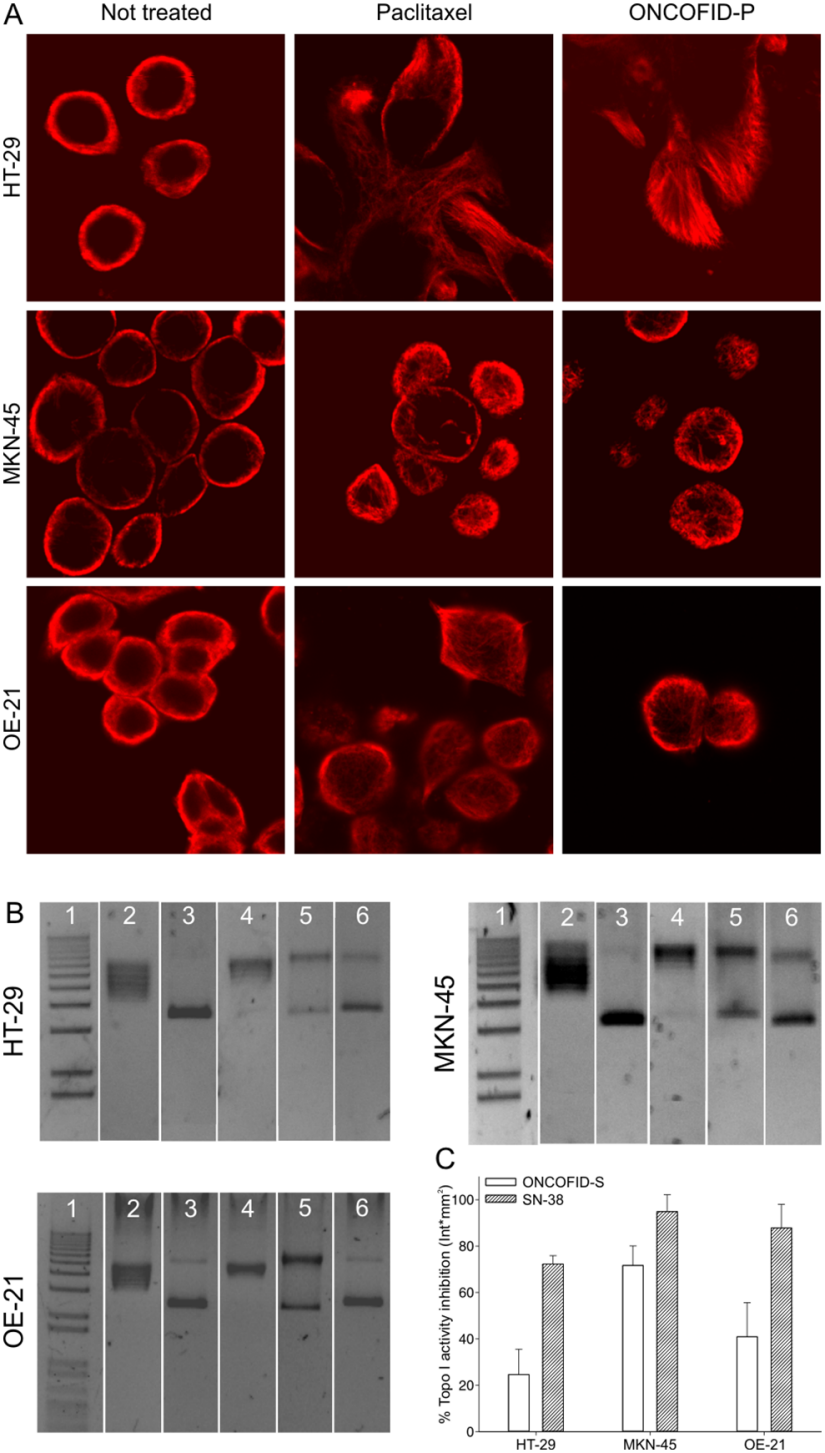
Assessment of bioconjugate mechanism of action. A, rearrangement of tumor cell microtubular architecture after drug treatment. HT-29, MKN-45 and OE-21 cells were treated with ONCOFID-P or free paclitaxel for 4 hours at 37°C. After treatment, cells were fixed, permeabilized, and stained with an anti-β-tubulin mAb and anti-mouse Ig Alexa 546-conjugated antiserum. Cells treated with free drug or bioconjugate disclosed the same interferences on the microtubular mesh. B, inhibition of Topo I activity after ONCOFID-S or SN-38 treatment in HT-29, MKN-45 and OE-21 cells. Gels show the supercoiled or relaxed forms of pBR322 plasmid after incubation with a 1∶50 dilution of nuclear protein neat extracts obtained from tumor cells treated with conjugated or free drug for 4 hours. Lane 1, marker; lane 2, relaxed pBR322 plasmid (positive control); lane 3, supercoiled plasmid (negative control); lane 4, supercoiled plasmid in the presence of nuclear protein neat extract from drug-untreated cells; lane 5, supercoiled pBR322 admixed with nuclear protein neat extract from ONCOFID-S treated cells; lane 6, supercoiled pBR322 admixed with nuclear protein neat extract from SN-38-treated cells. C, quantification of the reactions shown in B. Figure reports mean ± SD of 3 independent experiments.

Paclitaxel profoundly interferes with the intracellular microtubular mesh. To evaluate the effects of ONCOFID-P on the cytoskeleton architecture, target cell lines were incubated with the bioconjugate or the free drug, and stained with an anti-β-tubulin mAb to be finally imaged by confocal microscopy. Results showed that the bioconjugate led to the formation of tubulin bundles similar to those induced by treatment with free drug (Fig. 4A), thus suggesting that the paclitaxel released by ONCOFID-P fully retains the capacity of interfering with the microtubule polymerization dynamics.

After being produced from irinotecan, SN-38 blocks the activity of Topo I, a nuclear enzyme which makes single-strand cuts in DNA to favor relaxation before cell duplication. To assess the effects of ONCOFID-S or the free drug on Topo I activity, nuclear protein extracts from treated cells were incubated with a plasmid DNA and the ratio between the supercoiled and relaxed forms was visualized (Fig. 4B) and quantified (Fig. 4C) by gel electrophoresis. Both ONCOFID-S and SN-38 inhibited the enzyme activity, even though the free drug appeared more efficient in particular in the colorectal and esophageal histotypes. Nonetheless, results suggest that active SN-38 molecules are released by the bioconjugate and have access to the nucleus where they can block Topo I activity.

### Evaluation of *in vitro* tumor growth inhibition activity of bioconjugates

To test ONCOFID-P and ONCOFID-S *in vitro* efficacy against target cancer lines, cells were incubated with escalating concentrations of bioconjugates and the resulting dose-dependent growth inhibition activity was compared to that exerted by the commercial free drugs. The antiproliferative activity of bioconjugates turned out to be comparable to that observed with the unconjugated drugs (Table S1); as expected, the inhibitory activity of irinotecan was limited because such drug requires *in vivo* activation and conversion [44]. No toxic effects could be ascribed to HA (data not shown).

As CD44 appears critically involved in binding HA-conjugated drugs (Fig. S3 and [40]), we further addressed the role of CD44 in conjugate binding and activity by isolating two HCT-15 sublines expressing respectively high and low levels of the receptor, to test their sensitivity to bioconjugate cytotoxicity respect to the parental cell line. Using either ONCOFID-P or ONCOFID-S, almost a one-log differential susceptibility was observed between HCT-15 sublines (Fig. S5). The total cell population did not display a perfectly intermediate behaviour between CD44^high^ and CD44^l^°^w^ cells, likely due to a receptor expression very close to that of the CD44^l^°^w^ subline. As controls, both HCT-15 sublines and parental cells exhibited the same sensitivity to the unconjugated drugs (Fig. S5, left panels).

### *In vivo* therapeutic activity of ONCOFID-P and ONCOFID-S

To test the therapeutic efficacy of bioconjugates in a PC context, models of diffuse carcinomatosis were set up for each tumor histotype under investigation by i.p. injection of MKN-45, HT-29 and OE-21 tumor cell lines. Pharmacological treatments were started at day 7 from tumor injection and carried out according to a q7dx3 schedule. In each experiment, groups of mice were injected with either ONCOFID-P or ONCOFID-S i.p., or the free drugs administered through the i.p. or i.v. routes for comparison. The low water solubility and high side toxicity precluded the use of free SN-38, which was then replaced by its precursor CPT-11, commonly used in the clinical setting. The therapeutic impact of the different approaches was assessed by luminescence, as the tumor cell lines tested had been previously transduced with a lentiviral vector coding for the firefly luciferase reporter gene to track tumor growth, and by recording survival.

**Figure 5:**
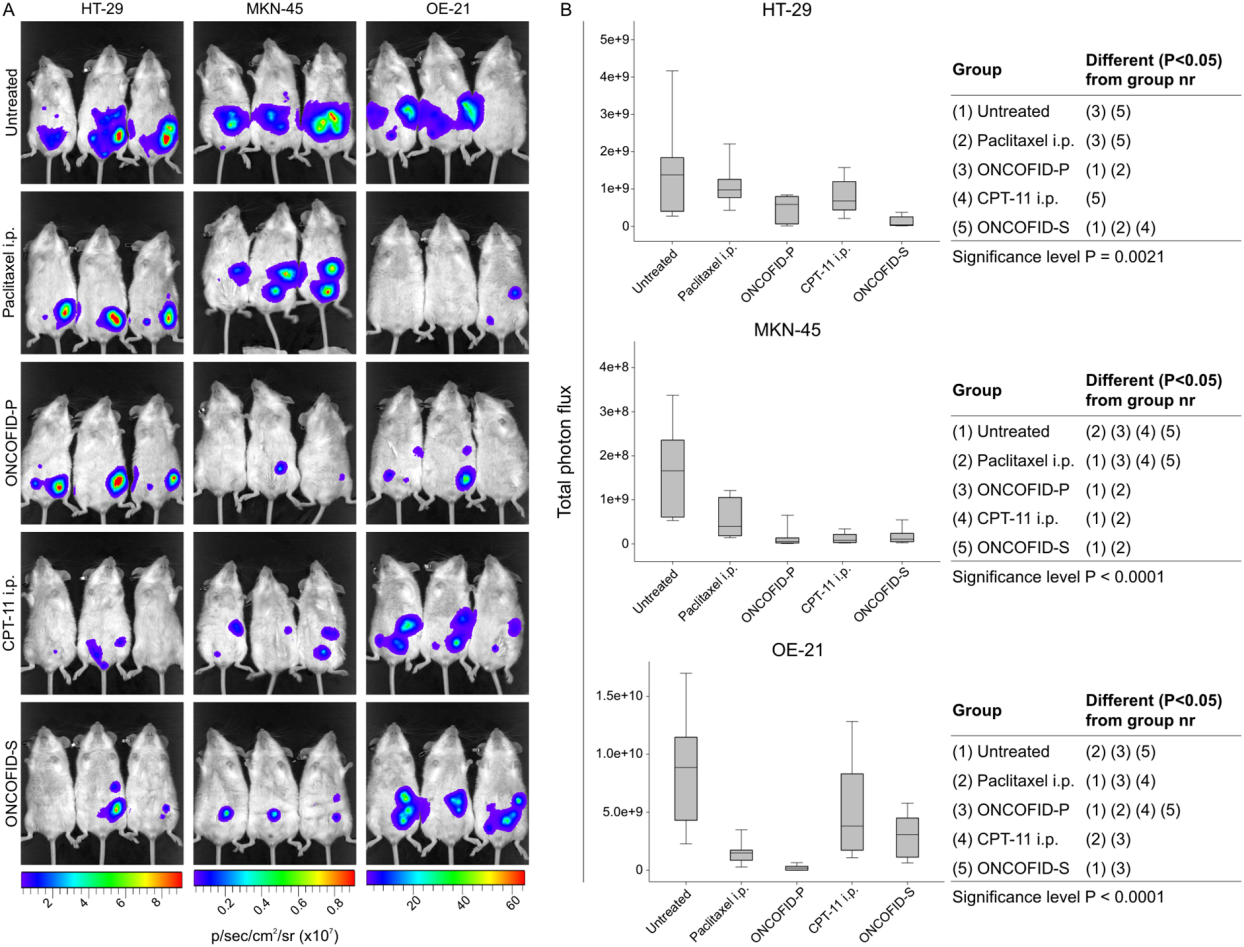
Assessment of *in vivo* tumor growth and response to therapy. A, bioluminescence imaging of pharmacologically treated or untreated mice with peritoneal carcinomatosis induced by luciferase-transduced tumors. Panels show three representative mice per group at one month after tumor injection. B, cumulative results. Each box plot reports mean ± SD of total photon emission from 6 mice per group at one month from peritoneal carcinomatosis induction. Statistical analysis (Kruskal-Wallis test) is reported in tables at the right of each panel.

As illustrated in Figure 5, ONCOFID-P loco-regional treatment brought about relevant therapeutic effects against all peritoneal carcinomatosis models, with a particular emphasis in gastric and esophageal cancers. When such results were compared to those obtained using the free drug given through different administration routes, it turned out that free paclitaxel given i.p. exhibited a modest efficacy, being less efficient against gastric and esophageal cancer but not colon carcinoma, and with a significant reduced activity compared to the conjugated form. On the other hand, the same free drug given i.v. was slightly more efficient in mediating antitumor effects, but not superior to ONCOFID-P (data not shown). Notably, a relevant tumor growth inhibition was also obtained with ONCOFID-S.

**Figure 6:**
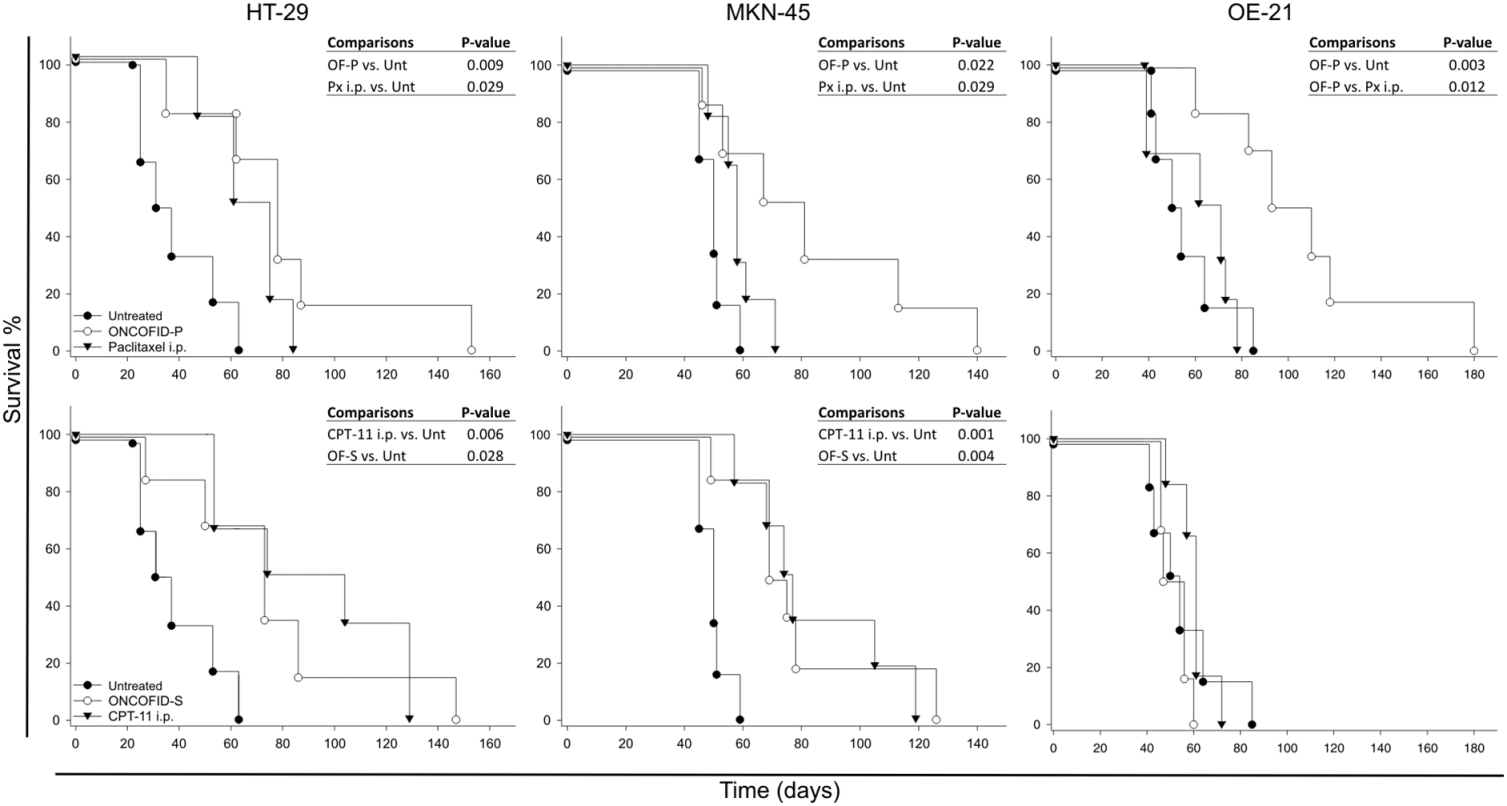
*In vivo* therapeutic activity of bioconjugates. Kaplan-Meier survival curves of mice with peritoneal carcinomatosis from HT-29, MKN-45 or OE-21 tumor cells. Animals were randomly assigned to an experimental group and drug treatment was initiated according to therapeutic schedule reported in Materials and Methods. All experimental groups were statistically compared each other, but only significant values are reported in each panel.

These results were partially confirmed by survival analysis (Fig. 6 and Table S2). Indeed, ONCOFID-P treatment significantly prolonged survival in all tumor models compared to controls, a result comparable to that obtained with the free drug irrespective of i.v. or i.p. administration. A notable exception was represented by OE-21 tumor-bearing mice, where the bioconjugate performed significantly better than the free drug. Similarly, ONCOFID-S exerted an important therapeutic activity against colon carcinoma and gastric, but not esophageal, peritoneal carcinomatosis, thus performing equally to the free CPT-11 drug administered either i.v. or i.p.

## Discussion

Peritoneal carcinomatosis is a severe condition often representing the final and fatal evolution of tumors arising in the gastrointestinal tract. However, the anatomical characteristics of the peritoneum make a loco-regional treatment an attractive and valid option to the systemic chemotherapy, which is widely used as support and palliative care. Indeed, in the last two decades cytoreductive surgery [45] followed by intraperitoneal chemotherapy (with or without associated hyperthermia) has increased the patients survival [46], [47].

However, in addition to drug-related adverse effects (in particular, bone marrow suppression [45]), these procedures are invasive, impact negatively the quality of life of patients [48], are not well standardized and also require high degree of specialization, since related morbidity and mortality rates (ranging from 25 to 41% and from 0 to 8%, respectively [49]) are critically lower in centers of expertise [9]. Other concerns arise from the limited choice of pharmacological loco-regional treatments, which is primarily dictated by the histotype of the original tumor and is presently based on the off-label use of i.v. drug formulations characterized by unsatisfactory pharmacokinetics and pharmacodynamics properties [50]. To the best of our knowledge, to date only catumomaxomab obtained the FDA approval for malignant ascites treatment [50]. Thus, *ad hoc* drug delivery strategies are focused on achieving higher local concentration of the drugs with a higher retention time in the cavity, thus reducing systemic adverse effects and local toxicity. To this end, different formulations have been exploited involving microspheres, nanoparticles, liposomes, micelles, implants and injectable depots. Nonetheless, each approach presents some drawbacks regarding the retention time, tumor penetration capacity, induction of inflammatory reactions or technical difficulties, as recently reviewed [51].

In such context, the use of hyaluronan-based bioconjugates can potentially overcome these issues. Based on the promising results obtained with the loco-regional use of ONCOFID-P [21], [33], [40], [52] and ONCOFID-S [11], we propose the administration of these bioconjugates for the treatment of peritoneal carcinomatosis. In addition to its well-known biocompatibility, hyaluronan was chosen because of its capability to selectively target tumor cells through the binding to CD44. This receptor is over-expressed in a wide variety of cancers, including tumor histotypes reported in this study [11], [28], [29].

Indeed, in addition to the results from competition experiments with anti-CD44 blocking mAb, the selectivity of these bioconjugates was confirmed by the fact that a CD44^l^°^w^ human colon cancer subline disclosed a differential susceptibility to ONCOFID-P and to ONCOFID-S respect to the related CD44^high^ counterpart. While conferring this selectivity, the chemical linking of paclitaxel and SN-38 to hyaluronan did not impact their biological effects, as demonstrated *in vitro* by the assessment of the microtubular structure alterations and the Topo I activity inhibition, respectively. This in turn reflects on biological activity *in vitro* and also *in vivo*, both shortly after the completion of the drug administration schedule (in terms of tumor growth as assessed by imaging), and long-term when considering survival. ONCOFID-P significantly reduced the short-term tumor burden in all analysed histotypes respect to the free drug; similar results were obtained for ONCOFID-S. Interestingly, ONCOFID-P performed equally to ONCOFID-S in both colorectal and gastric carcinomatosis. In this regard, it should be noted that the conjugation with HA allows the successful employment of paclitaxel against colorectal carcinoma, an use that has not a FDA approval and consideration in the clinical setting.

In long-term analysis, ONCOFID-P improved therapeutic outcome of esophageal peritoneal carcinomatosis respect to the related free drugs; moreover, an increase in median survival could be also observed against MKN-45 model, while not reaching statistical significance. In all other cases, the bioconjugates displayed encouraging results fully overlapping those achieved by the related free drugs. Nonetheless, the conjugation with HA brings about relevant advantages. From a pharmacological point of view, it increases the water solubility of paclitaxel, thus eliminating the adverse effects related to the currently used solvent Cremophor EL (inflammatory and hypersensitivity reactions, massive leukocyte infiltration involving both the mesothelial lining and the underlying muscle abdominal wall [33], neurotoxicity [53] and the need for long infusion time [54]). In addition, the conjugation with HA allows the administration of the CPT-11 active metabolite SN-38, which is at least 100 fold more active *in vitro* than CPT-11 at equimolar concentrations, but whose use is precluded due to its intrinsic toxicity and extremely low water solubility [11]. Moreover, it has been demonstrated that the hyaluronan *per se* reduces postoperative and disease-related adhesions without impacting the metastatic potential of tumor cells [55]. From a clinical point of view, the therapeutic outcome is achieved with negligible adverse effects. Indeed, we and others [33], [11] previously reported that both bioconjugates did not induce any sign of local toxicity, when administered at the same or even superior amounts. In addition, ONCOFID-S does not cause myelotoxicity, thus potentially representing an attractive candidate for the treatment of UGTA1 genotype patients, which are predisposed to develop severe neutropenia related to CPT-11 [11]. The virtually absence of critical side effects and the specific tumor targeting allow the use of increased administration doses (a 4- and 3-times increase for ONCOFID-P *vs* paclitaxel i.p. and for ONCOFID-S *vs* CPT-11 i.p. in SN-38 equivalents, respectively).

Moreover, it is worth noting that our results are mainly obtained with drugs used for off-label indications, administered only anecdotally against the tumor histotypes under study. In particular, as for short-term tumor growth analysis, the treatment of colorectal carcinoma with ONCOFID-P improves mice survival at a comparable extent to ONCOFID-S, which carry the on-label, widely used CPT-11 metabolite.

In conclusion, these data corroborate previously successful results in the management of bladder and ovarian cancer, and envisage that the conjugation with HA can widen the use of existing drugs over their formal approval or current use, thus providing a strategy to potentially improve the loco-regional treatment of peritoneal carcinomatosis.

## Supporting Information

Figure S1 **CD44 and intracellular RHAMM expression in different tumor cell lines.** A, viable cells were stained with a FITC-labeled anti-human CD44 mAb. B, fixed and permeabilized cells were stained with an anti-human CD168 mAb followed by an Alexa 546-conjugated anti-Ig mouse serum. In both panels A and B, insets show flow cytometry analysis of HCT-15 parental cell line.
(TIF)

Figure S2 **Kinetics of interaction between ONCOFID-P and tumor cell lines, in the presence of hyaluronidase.** BODIPY-labeled ONCOFID-P was added to tumor cells for different time points (0.5, 1, 2, 5, 10 and 15 minutes); after extensive washing, samples were added with hyaluronidase for 4 hours or left untreated, and flow cytometry analysis was finally performed. A, whole kinetics of interaction at all time points tested. B, kinetics of the fluorescence intensity (geo mean) detected on tumor cells at the same time points analyzed as in A. Panels A and B report mean ± SD of 3 independent experiments.
(TIF)

Figure S3 **Blocking of the ONCOFID-P-receptor interaction by an anti-CD44 antibody.** HT-29, MKN-45 and OE-21 tumor cells were incubated with BODIPY-labeled ONCOFID-P alone (solid line) or in the presence of an anti-CD44 blocking mAb (dashed line), and analyzed by flow cytometry. Data at the upper-right corner of each panel report the respective geo mean values and the percentage of reduction induced by anti-CD44 mAb blocking treatment.
(TIF)

Figure S4 **BODIPY labeling does not alter ONCOFID-S activity.** Representative tumor cell lines were incubated with escalating concentrations of unlabeled and BODIPY-labeled ONCOFID-S, and the resulting growth inhibition was evaluated by ATPlite assay. Unlabeled and labeled bioconjugates showed fully overlapping dose-response curves. The values of IC_50_ reported were calculated from these semi-logarithmic dose-response curves by linear interpolation.
(TIF)

Figure S5 **Impact of differential CD44 expression on bioconjugate cytotoxic activity.** The parental (HCT-15) and the selected CD44^l^°^w^ (HCT-15 CD44^l^°^w^) and CD44^high^ (HCT-15 CD44^high^) HCT-15 colorectal cell lines were incubated with escalating doses of paclitaxel (upper left panel), ONCOFID-P (upper right panel), SN-38 (lower left panel) and ONCOFID-S (lower right panel). The resulting growth inhibition was evaluated by ATPlite assay. Figure shows mean ± SD of three independent experiments. Extrapolated IC_50_ values are reported in pg/mL in free drug equivalents.
(TIF)

Table S1 **IC_50_ values of free and HA-conjugated drugs.** The values of the reported IC_50_ are the mean ± SE of five viability experiments carried out for each tumor cell line. For each experiment, the IC_50_ was calculated from each single semi-logarithmic dose-response curve by linear interpolation, and obtained values were then averaged. Values are reported in ng/mL and for each bioconjugate they are expressed in terms of free drug equivalents. No significant difference is evidenced between the activities of HA-bound and free drugs (Mann-Whitney Rank Sum Test).
(XLSX)

Table S2 **Median survival of tumor-bearing mice treated with free and conjugated drugs.**
(XLSX)
