# Supplementary figures and images for: Peritoneal Tumor Carcinomatosis: Pharmacological Targeting with Hyaluronan-Based Bioconjugates Overcomes Therapeutic Indications of Current Drugs
Source: PLoS One. 2014 Nov 10;9(11):e112240. doi: 10.1371/journal.pone.0112240 (PMC4226476; doi:10.1371/journal.pone.0112240)

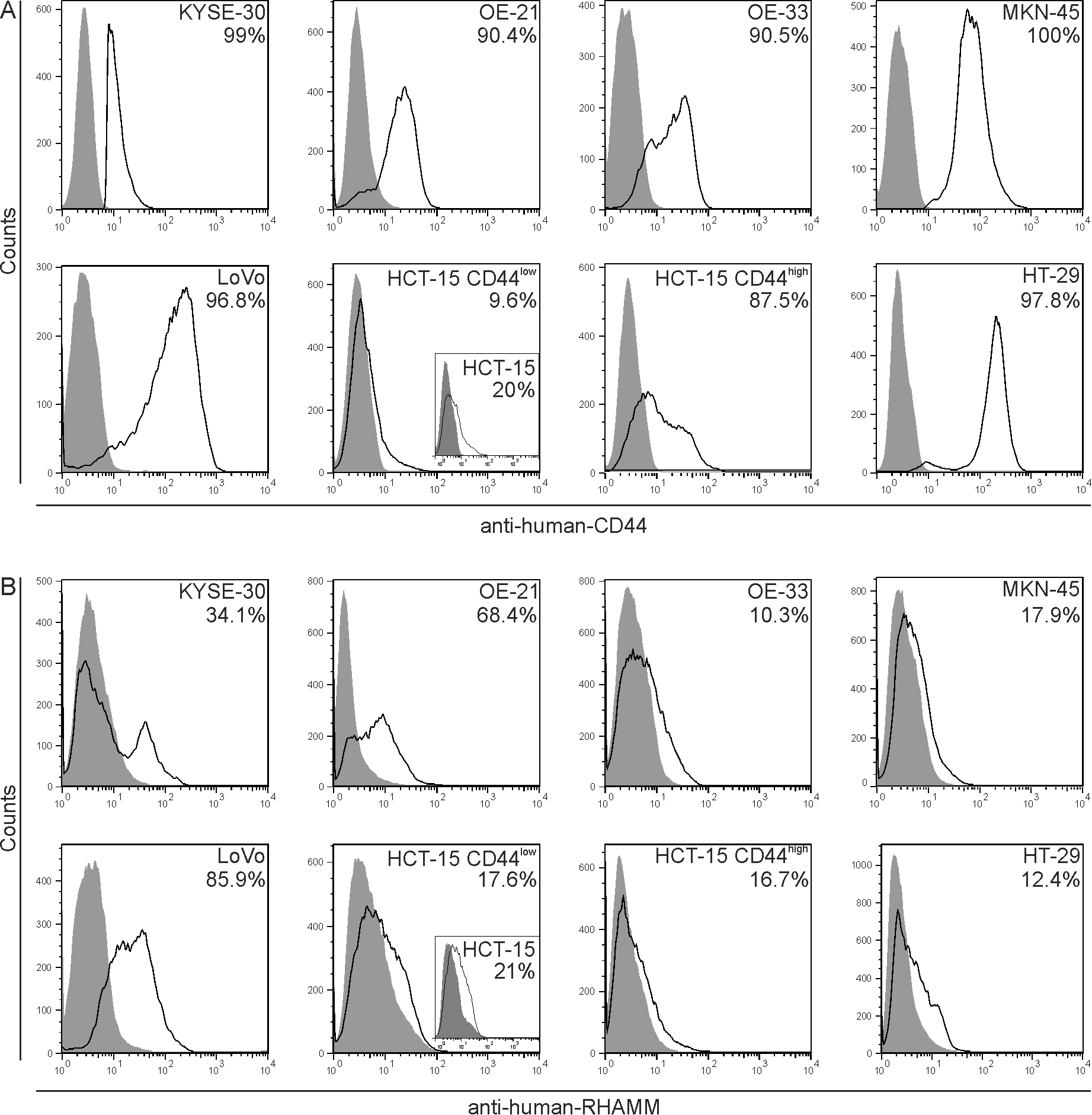

Supplement: Figure S1 — CD44 and intracellular RHAMM expression in different tumor cell lines. A, viable cells were stained with a FITC-labeled anti-human CD44 mAb. B, fixed and permeabilized cells were stained with an anti-human CD168 mAb followed by an Alexa 546-conjugated anti-Ig mouse serum. In both panels A and B, insets show flow cytometry analysis of HCT-15 parental cell line. (TIF) [file pone.0112240.s001.tif]

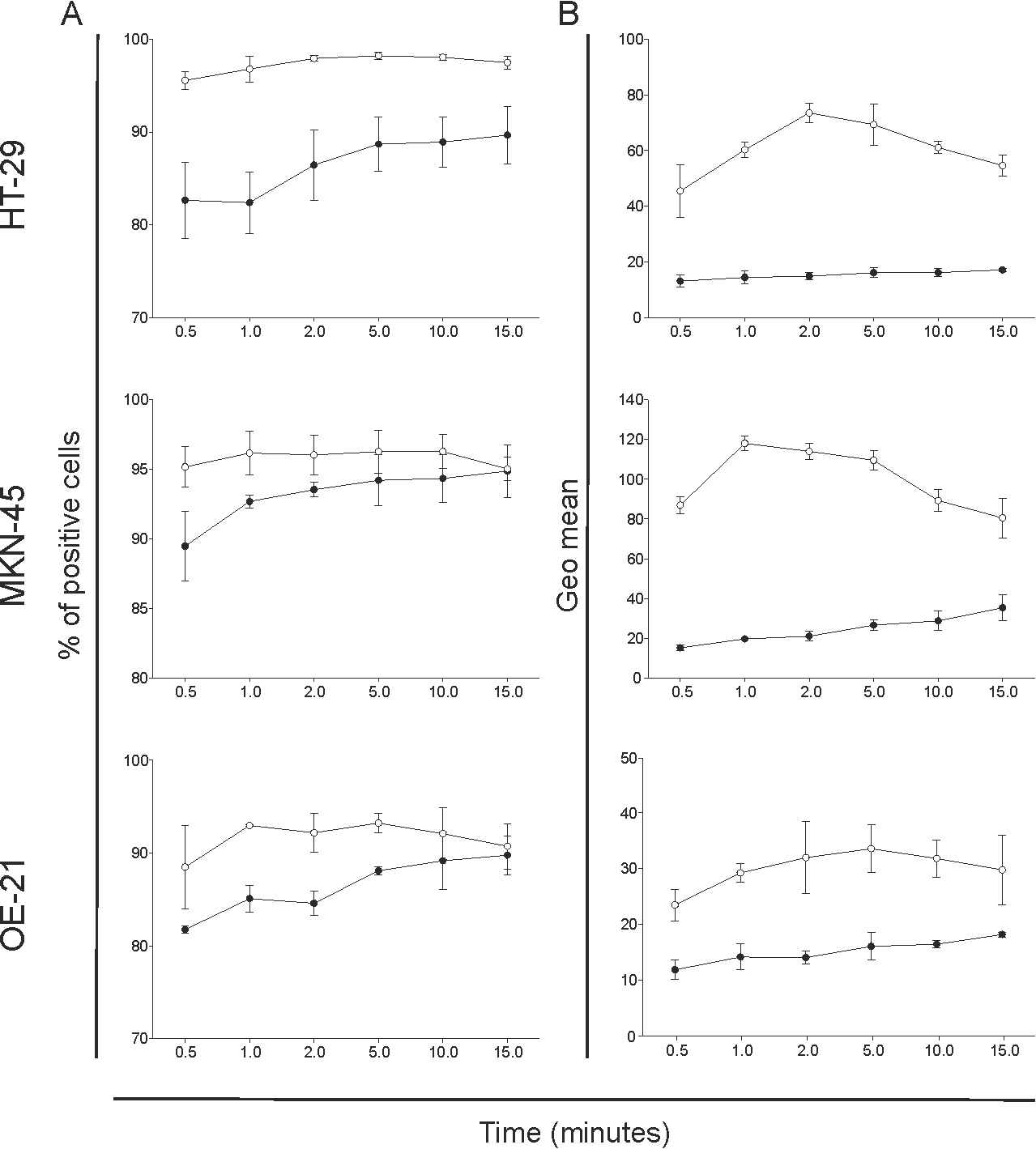

Supplement: Figure S2 — Kinetics of interaction between ONCOFID-P and tumor cell lines, in the presence of hyaluronidase. BODIPY-labeled ONCOFID-P was added to tumor cells for different time points (0.5, 1, 2, 5, 10 and 15 minutes); after extensive washing, samples were added with hyaluronidase for 4 hours or left untreated, and flow cytometry analysis was finally performed. A, whole kinetics of interaction at all time points tested. B, kinetics of the fluorescence intensity (geo mean) detected on tumor cells at the same time points analyzed as in A. Panels A and B report mean ± SD of 3 independent experiments. (TIF) [file pone.0112240.s002.tif]

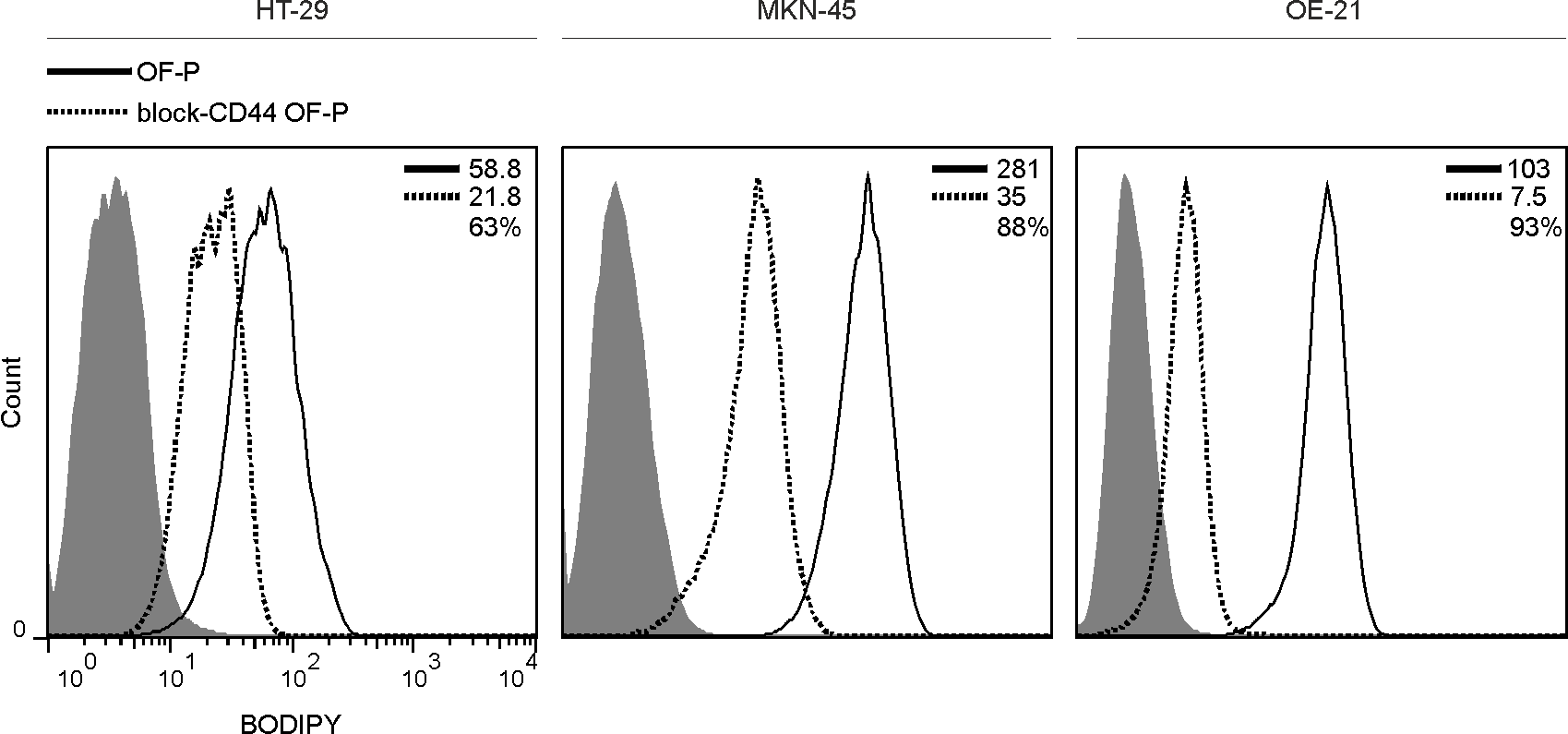

Supplement: Figure S3 — Blocking of the ONCOFID-P-receptor interaction by an anti-CD44 antibody. HT-29, MKN-45 and OE-21 tumor cells were incubated with BODIPY-labeled ONCOFID-P alone (solid line) or in the presence of an anti-CD44 blocking mAb (dashed line), and analyzed by flow cytometry. Data at the upper-right corner of each panel report the respective geo mean values and the percentage of reduction induced by anti-CD44 mAb blocking treatment. (TIF) [file pone.0112240.s003.tif]

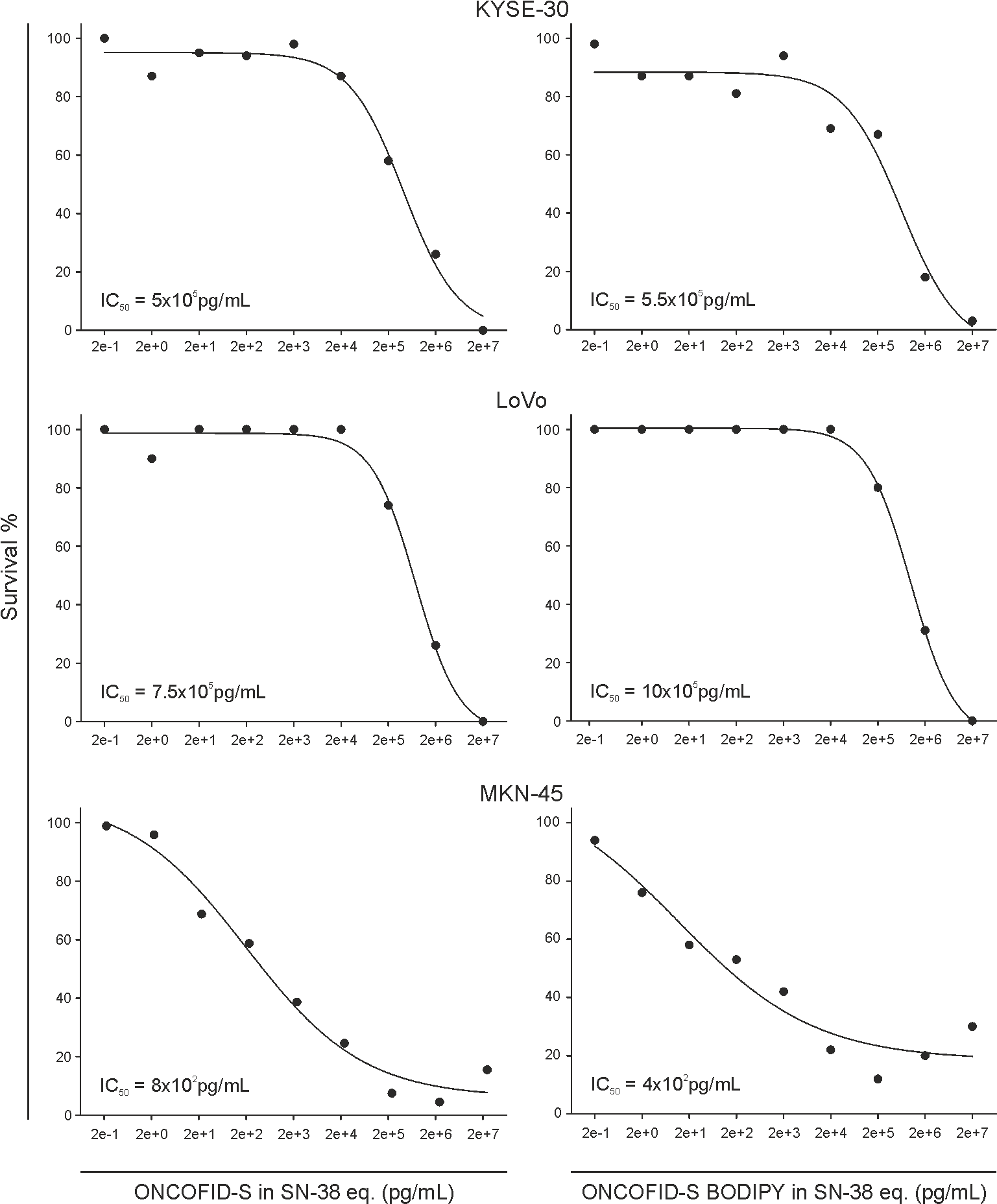

Supplement: Figure S4 — BODIPY labeling does not alter ONCOFID-S activity. Representative tumor cell lines were incubated with escalating concentrations of unlabeled and BODIPY-labeled ONCOFID-S, and the resulting growth inhibition was evaluated by ATPlite assay. Unlabeled and labeled bioconjugates showed fully overlapping dose-response curves. The values of IC50 reported were calculated from these semi-logarithmic dose-response curves by linear interpolation. (TIF) [file pone.0112240.s004.tif]

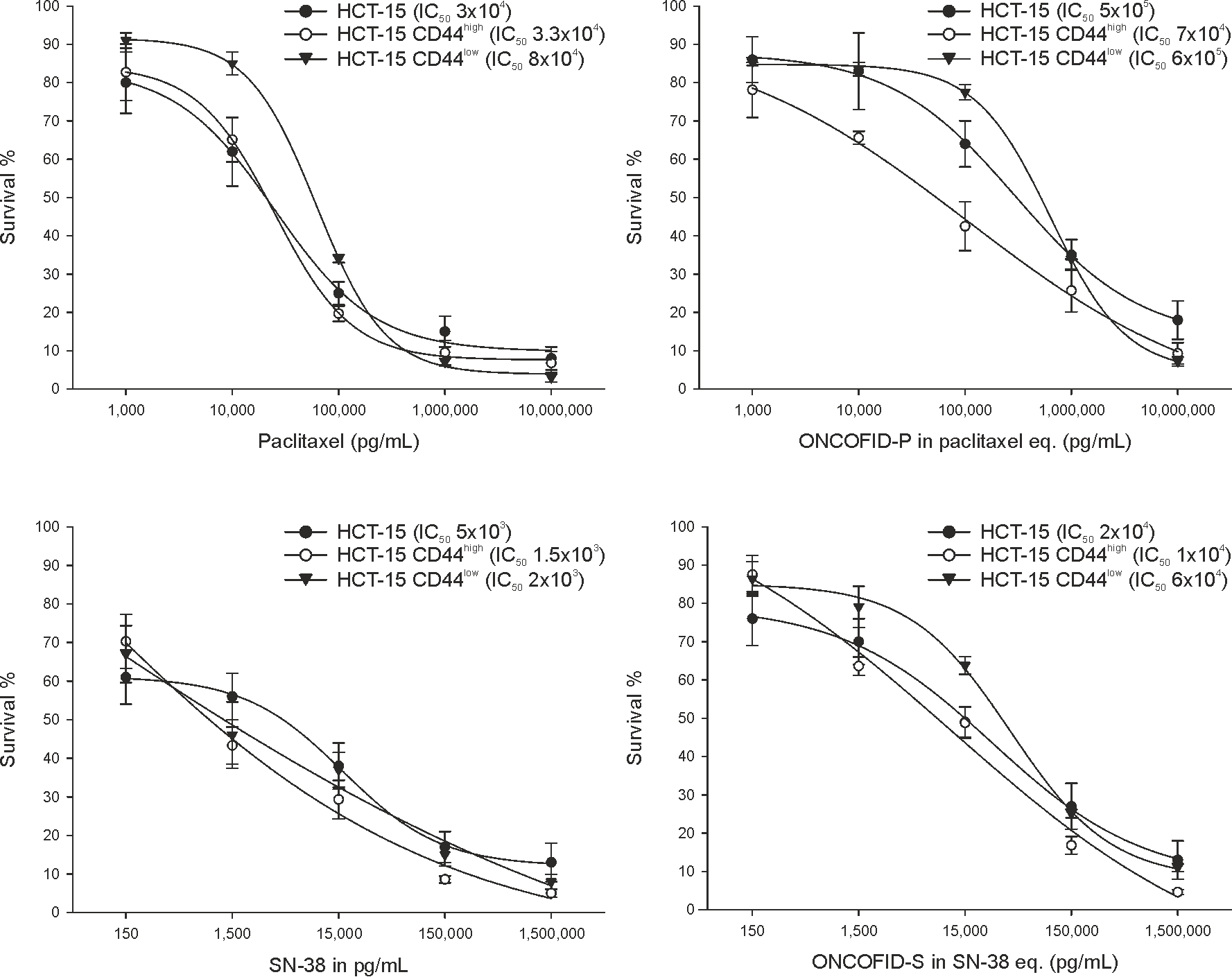

Supplement: Figure S5 — Impact of differential CD44 expression on bioconjugate cytotoxic activity. The parental (HCT-15) and the selected CD44l°w (HCT-15 CD44l°w) and CD44high (HCT-15 CD44high) HCT-15 colorectal cell lines were incubated with escalating doses of paclitaxel (upper left panel), ONCOFID-P (upper right panel), SN-38 (lower left panel) and ONCOFID-S (lower right panel). The resulting growth inhibition was evaluated by ATPlite assay. Figure shows mean ± SD of three independent experiments. Extrapolated IC50 values are reported in pg/mL in free drug equivalents. (TIF) [file pone.0112240.s005.tif]
